# Neuroanatomy- and Pathology-Based Functional Examinations of Experimental Stroke in Rats: Development and Validation of a New Behavioral Scoring System

**DOI:** 10.3389/fnbeh.2018.00316

**Published:** 2018-12-18

**Authors:** Shin-Joe Yeh, Sung-Chun Tang, Li-Kai Tsai, Jiann-Shing Jeng, Chi-Ling Chen, Sung-Tsang Hsieh

**Affiliations:** ^1^Graduate Institute of Anatomy and Cell Biology, College of Medicine, National Taiwan University, Taipei, Taiwan; ^2^Department of Neurology and Stroke Center, National Taiwan University Hospital, Taipei, Taiwan; ^3^Graduate Institute of Clinical Medicine, College of Medicine, National Taiwan University, Taipei, Taiwan; ^4^Graduate Institute of Brain and Mind Sciences, College of Medicine, National Taiwan University, Taipei, Taiwan; ^5^Center of Precision Medicine, College of Medicine, National Taiwan University, Taipei, Taiwan

**Keywords:** stroke, neuroanatomy, functional examinations, garcia score, circling, infarct size

## Abstract

In experimental stroke studies, a neuroanatomy-based functional examination of behaviors is critical to predict the pathological extent of infarcts because brain-imaging studies are not always available. However, there is a lack of systematic studies to examine the efficiency of a behavioral test for this purpose. Our work aimed to design a new score for this goal in stroke rats, by simplifying the Garcia score (with subscore 1–6) and adding circling as subscore 7. MRI and 2,3,5-triphenyltetrazolium chloride staining were used to determine the pathological extent after transient middle cerebral artery occlusion. The modified summations of subscores were designed according to the predictability of each subscore for locations and sizes of infarcts in one group of stroke rats, and were validated in another group. The original Garcia score was able to predict the pathological extent of edema-adjusted infarct size ≥30%, and the summation of subscore 4, 6, and 7 (4: climbing, 6: vibrissae sensation, 7: circling) also could predict it well. The original Garcia score failed to predict infarct at the primary motor cortex, while the summation of subscore 4, 6, and 7 potentially could predict not only the primary motor cortex, but also the forelimb, hindlimb, and barrel field regions of the primary sensory cortex. Accordingly, this neuroanatomy-correlated functional assessment system composed of subscore 4, 6, and 7 was proposed, with less examination time and better inter-rater reliability than the original Garcia score. In summary, this new scoring system, summation (4,6,7) score, examined motor and sensory functions based on neuroanatomical involvement, having the potential to predict the pathological extent and specific relevant brain areas of infarcts, respectively.

## Introduction

Rodent stroke models are widely applied for investigating mechanisms and therapies of ischemic stroke (Fluri et al., 2015). Transient middle cerebral artery occlusion (tMCAO) model is commonly used to develop infarcts at basal ganglion and cerebral cortex (Ginsberg, 2003; Macrae, 2011). A critical issue in the assessments is accurate prediction of the pathological extent of infarcts, including the neuroanatomical involvement and size, before intervention. But the variability of the pathological extent in this model has become a significant confounder in the outcome evaluation (Takano et al., 1997). Clinically, neuroimaging with MRI or CT is a routine examination for human stroke. However, MRI scanning in animals is time-consuming, not always available, and takes a risk of respiratory or hemodynamic suppression due to prolonged anesthesia (Murakami et al., 2014). Therefore, accurate prediction of the pathological extent and neuroanatomy of infarcts by a method other than MRI is mandatory for outcome evaluation in the studies using rat stroke models.

Different brain regions exert distinct functions. Hence, behavioral tests potentially provide surrogate markers for assessing functional alterations due to structural changes after infarcts. Ideally, a behavioral test should fulfill the following requirements, such as reflecting the pathological extent and defined neuroanatomical areas of the infarcts, high inter-rater agreement, convenience to be performed, and short examination time. Most studies have used neurological behavioral assessments and infarct size evaluation as two independent parameters of outcome evaluation (Belayev et al., 1995; Luo et al., 2013; Yu et al., 2016; Gupta et al., 2017). Limited number of studies have reported the correlations between behavior scores and total infarct size or regional infarct size of one specific brain area (Supplementary Table 1) (Bederson et al., 1986; Grabowski et al., 1993; Yonemori et al., 1996; Rogers et al., 1997; Zausinger et al., 2000; Ishibashi et al., 2003, 2004; Wen et al., 2017). There is a lack of systematic studies on predicting specific brain regions and pathological extent of infarcts based on a structurally and functionally correlated behavioral test.

Examination time is another major limitation of behavioral tests. Task-specific tests are time-consuming partially because of the requirement of pre-training. Simple scoring systems do not require pre-training (Longa et al., 1989; Zhang et al., 2017), but they evaluate motor functions only. Composite scores have the advantages of incorporating different dimensions of neurological functions (Garcia et al., 1995; Shohami et al., 1995), but require more assessment time. Garcia score is one of the commonly used composite assessments encompassing motor, sensory, and exploratory behaviors (Garcia et al., 1995). Some studies used this score with minor modifications in the operational definitions but kept the original 6 subscores (Hartman et al., 2009; Jeon et al., 2009). Circling gait, a prominent gait pattern in stroke rats (Longa et al., 1989), is not included in this score. This study aimed to propose a neuroanatomy-based functional scoring system for rat stroke models by analyzing (1) the pathological significance of each subscore in the original Garcia score, correlated with infarct size and location, respectively, (2) the feasibility of including “assessment of circling” in the scoring system, and (3) the prediction ability of various combinations of these subscores to determine the sizes and locations of infarcts.

## Materials and Methods

### Ischemic Stroke Model of Rats

This study was carried out using an ischemia-reperfusion model of rats, by performing transient occlusion of the middle cerebral artery (tMCAO) with a silicon-coated nylon filament (MCAO sutures, RWD Life Science, Shenzhen, China) (Schmid-Elsaesser et al., 1998). Male *Sprague-Dawley* rats were used, supplied by BioLASCO Taiwan Co., with ages of 7–9 weeks old and body weight of 181–336 g. The sizes of silicon-coated filaments were chosen according to the body weight of each rat (181–235 g: silicon diameter 0.34 ± 0.02 mm, nylon diameter 0.24 mm; 235–270 g: silicon diameter 0.37 ± 0.02 mm, nylon diameter 0.28 mm; 270–336 g: silicon diameter 0.43 ± 0.02 mm, nylon diameter 0.28 mm). Different ischemic durations (0.5, 0.75, 1, 1.5, 2, and 3 h) were designed to develop a wide range of infarcts. Before the operation, the rats were supplied with water and food ad lib. Each rat was allocated randomly into one of these groups of different ischemic durations. Under inhalation anesthesia with isoflurane (induction dosage: 4%, maintenance dosage: 2%), anterior neck incision at the right paramedian line (5 mm from the midline) was performed to expose the right carotid artery. After serial ligations of the right proximal common carotid artery (CCA), external carotid artery, internal carotid artery (ICA), and with a loose tie at the distal CCA, a silicon-coated nylon filament was inserted through the right CCA and carefully advanced into the right ICA until light resistance developed. The rats were allowed to regain consciousness after fixation of the filament on ICA followed by closure of the neck wound (Virley et al., 2000). During ischemic period, the animals with circling behavior were included in this study to ensure successful occlusion of the middle cerebral artery. Near the end of ischemic period, the rats received anesthesia again for removal of the filament to achieve reperfusion. This surgical procedure minimized and standardized the exposure duration to isoflurane among different groups, in order to reduce the influence of neuroprotective effect of isoflurane on ischemic damage (Sakai et al., 2007; Taheri et al., 2014). The rats complicated with subarachnoid hemorrhage which was noted upon MRI examination or sacrifice were excluded. This study was carried out in accordance with the principles of the Basel Declaration. The protocol was approved by the Animal Committee of National Taiwan University College of Medicine.

### Study Design

A discovery group of stroke rats (*n* = 31) was used to explore the relationship between behavioral tests (Garcia score) and infarct size as well as infarcted neuroanatomical locations, and a modified Garcia score was proposed according to the summation of significant subscores. We applied another group of stroke rats (validation group, *n* = 42) for statistical validation of the original and modified Garcia scores. The pathological extent of infarct was evaluated in each rat in the two groups by 2,3,5-triphenyltetrazolium chloride (TTC) staining (Benedek et al., 2006), and 11 rats within the validation group received MRI examination before TTC staining for the sub-study of MRI-TTC correlation. To evaluate whether the scores would change according to different time windows, additional 10 stroke rats with ischemic duration 1.5 h were used to evaluate the behavioral tests serially at post-stroke 24, 72, and 120 h of each animal.

### Behavioral Tests

Behavioral tests were performed during daytime, at 24–96 h after initiation of reperfusion. The original Garcia score included 6 subscores (referred as subscore in this report): 1, spontaneous activity; 2, symmetry in the movement of four limbs; 3, forepaw outstretching; 4, climbing; 5, responses to touch of body (abbreviated as body sensation); 6, responses to touch of vibrissae (abbreviated as vibrissae sensation) (Garcia et al., 1995). We added a new subscore: 7, to assess the circling gait pattern, according to the grades as follows: 3, no circling in walking; 2, circling gait; and 1, unable to stand or walk. The operational definitions of all of the subscores of Garcia score and the new subscore 7 were shown in Table 1. Inter-rater reliability was tested in two observers, who were blinded to the ischemic conditions of the rats. The time taken for each subscore of the behavioral tests was also recorded.

**Table 1:** Operational definitions of the Garcia score (subscore 1–6) and the subscore of circling (subscore 7).

**Subscore**	**Description**	**3**	**2**	**1**	**0**
1.Spontaneous activity	Observation of the ability to approach the upper rims of all 4 walls of its own cage in 5 min	Approach the upper rims of 3–4 walls of the cage	Approach the upper rims of 1–2 walls of the cage	Not rise up and barely move	Not move at all
2.Symmetry in the movement of four limbs	Holding the rat in the air by the tail to observe for symmetry in the movement of the four limbs	All four limbs extend symmetrically	One forelimb extends less or more slowly than the other side	One forelimb has minimal movement	One forelimb does not move
3.Forepaw outstretching	Walking on forelimbs while the rat is held by the tail	Both forelimbs are outstretched, and the rat walks symmetrically	One side outstretches less than the other, or deviation in walking	One forelimb has minimal movement	One forelimb does not move
4.Climbing	Observation in 3 trials for reaching the top of the grid wall^*^ and the symmetry of gripping power of bilateral forelimbs	Reach the top in 3 trials with symmetric gripping power	Reach the top in 1–2 trials, using wrist instead of hand in holding the grid, trunk tilting during climbing, or asymmetric gripping power	Failed to climb to the top, or tend to circle instead of climb	
5.Body sensation	Observation of the response to touch on each side of the lower part of body with a blunt stick	Turn head to the stimulus and is equally startled by the stimulus on both sides	React slowly to stimulus on one side	Not respond to the stimulus on one side	
6.Vibrissae touch	Observation of the reaction to vibrissae touch on each side by a thin stick	Symmetric response of turning head to stimuli or startle response	Slower response on one side	No response on one side	
7.Circling	Observation of circling in spontaneous gait in an open field^†^	No circling in walking	Circling gait	Unable to stand or walk	

* *Grid wall: The size of each grid is 2.5 × 2.5 cm, and the size of the grid wall is 25 cm in width and 35 cm in height*.

† *Open field: The size is 1 meter in length and width*.

*Order in performing these subscores was 1, 5, 6, 7, 2, 3, 4 (The order was arranged according to the disturbance to rats: from low to high disturbance)*.

### Quantification of Infarct Size and Identification of Infarct Location

In the MRI-TTC correlation sub-study, 11 stroke rats were used with ischemic durations of 0.75 h (*n* = 3), 1 h (*n* = 6), and 1.5 h (*n* = 2) to induce a wide range of infarct size. Since this sub-study focused on the relationship between the infarct sizes on MRI and TTC, ischemic duration was an indirect contributor in this correlational analysis. The brain MR images were acquired using 7T MRI (Bruker PharmaScan, Ettlingen, Germany) at National Taiwan University, Taipei, Taiwan. The parameters of the diffusion-weighted imaging (DWI) sequence were as follows (Tsai et al., 2011): *b*-value 1,000 s/mm^2^, repetition time (TR) 4,500 ms, echo time (TE) 30 ms, section thickness 1 mm, field of view 2.56 × 2.56 cm, and matrix size 128 × 128. The parameters of T2-weighted imaging were as follows: 15 contiguous, coronal slices (thickness: 1 mm) were acquired with a field of view of 2.56 × 2.56 cm, matrix size 256 × 256, TR 3,000 ms, and TE 50 ms. The MR images were analyzed with ImageJ software (National Institutes of Health, Bethesda, Maryland, USA).

After behavioral evaluation and MRI scanning, the rats were sacrificed for *in vitro* TTC staining. The colorless TTC is reduced to a red formazan product by dehydrogenase abundant in mitochondria of non-ischemic tissue, while the ischemic tissue remains white under the staining, allowing for the differentiation between ischemic and non-ischemic tissue macroscopically (Benedek et al., 2006). The stained sections were coded, photographed, and then analyzed by the examiner blinded to the coded information. The digital images were analyzed by outlining the infarct area in ImageJ software (National Institutes of Health, Bethesda, MD). Direct infarct volume [(summation of infarct areas of the 8 slices) × thickness 2 mm] and direct infarct ratio (direct infarct volume/contralateral hemisphere volume) were calculated. Edema-adjusted infarct volume was calculated as “contralateral hemisphere volume—ipsilateral non-infarcted volume” (Lin et al., 1993). Edema-adjusted infarct size (%) was defined as edema-adjusted infarct volume divided by contralateral hemisphere volume. Using ratios instead of absolute volumes to present infarct sizes in this study except for the MRI-TTC correlation sub-study was to preclude the potential confounding effect of body weight to the brain volume (Bailey et al., 2004). Four neuroanatomical areas were identified in infarct regions according to a rat-brain atlas (Paxinos and Watson, 2007), including the primary motor cortex (M1), the forelimb region of the primary sensory cortex (S1FL), the hindlimb region of the primary sensory cortex (S1HL), and the barrel field region of the primary sensory cortex (S1BF). These 4 areas were selected based on potential functional correlations with the Garcia subscores.

### Statistical Analysis

First, the relationship between infarct size (with/without edema-adjustment) and ischemic duration was analyzed. The infarct sizes of different ischemic durations were compared by Kruskal-Wallis test. Edema-adjusted infarct size and direct infarct size within the same ischemic duration were compared by Wilcoxon matched-pairs signed-rank test. Second, for predicting infarct size or specific neuroanatomical location by the subscores or by the modified summations of subscores obtained by different combinations of subscores, logistic regression analysis was used. Edema-adjusted infarct size ≥30% was a determined cut-off point because the average infarct size in this stroke model of *Sprague-Dawley* rats is around 30% (Conn, 2017). Since ischemic duration was a significant determinant of infarct volume (Fluri et al., 2015), this parameter was adjusted in the logistic regression models. Modified summations of subscores were designed according to the correlation of each subscore with infarct size or location in a discovery group of stroke rats, respectively. We measured area under receiver operating characteristic curve (AUC) in each modified combination to compare its prediction ability with that of the original Garcia score. For predicting infarct in M1, S1FL, S1HL, and S1BF, their correlations with each subscore and different combinations of subscores were analyzed. Third, the Garcia scores between different time points of 24, 72, and 120 h were compared with each other by Wilcoxon matched-pairs signed-rank test, and so was the modified Garcia score.

Fourth, to validate the modified Garcia score, we applied another group of stroke rats (validation group) to check for the predictability of the modified score for infarct size or infarct neuroanatomical locations, analyzing the sensitivity, specificity, positive predictive value, and negative predictive value, respectively, which were compared with the original Garcia score by McNemar test. Fifth, in the validation group, we compared the modified Garcia scores in the rats with infarct in one specific location to those without infarct in the same region. Sixth, Kappa statistic was calculated for inter-rater reliability between two independent observers for the original Garcia score and the modified Garcia score, respectively, in 13 rats of tMCAO (4 rats from the validation group and 9 rats from an additional group). The total examination time for the modified Garcia score in the aforementioned 13 rats was also compared with that of the original Garcia score by Mann-Whitney *U* test. Stata software (StataCorp LLC, Texas, USA) and IBM SPSS Statistics (IBM, New York, USA) were applied for these statistical analyses.

## Results

### Relationship Between MRI and TTC Staining

**Figure 1:**
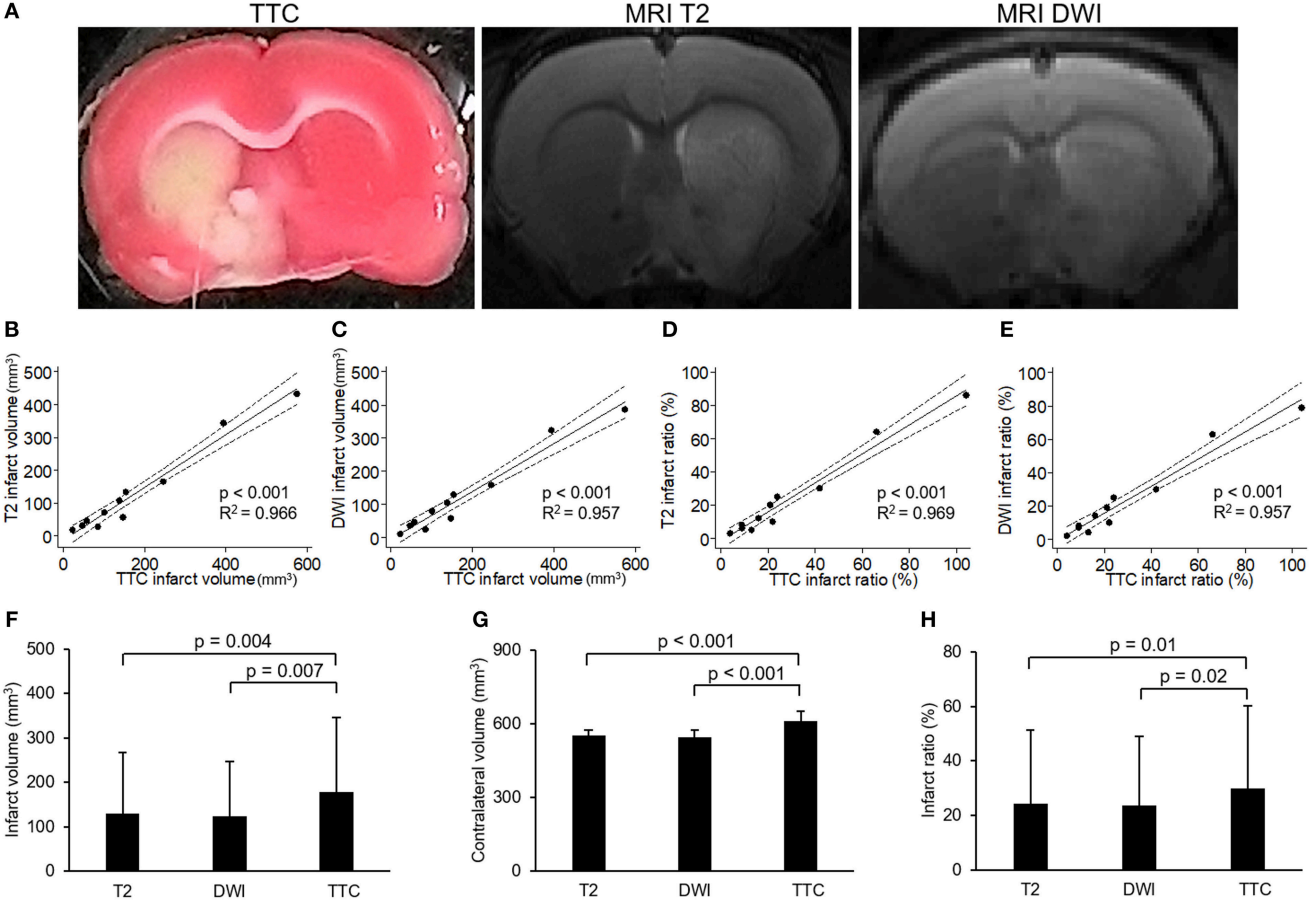
Correlation between images of 2,3,5-triphenyltetrazolium chloride (TTC) staining and MR. **(A)** Example of images of 2,3,5-triphenyltetrazolium chloride (TTC) staining and MR (T2 and diffusion weighted image [DWI]) at the level of anterior commissure of a rat brain after transient middle cerebral artery occlusion. **(B–E)** showed the results of linear regression model for the correlations of infarct volumes or infarct ratios between TTC images and MRI (solid line: fit line of this model; dotted line: 95% confidence interval): **(B)** Correlation of infarct volumes between TTC images and T2. **(C)** Correlation of infarct volumes between TTC images and DWI. **(D)** Correlation of infarct ratios between TTC images and T2 (infarct ratio = infarct volume/contralateral hemisphere volume). **(E)** Correlation of infarct ratios between TTC images and DWI. **(F–H)** showed the results of comparisons of infarct volumes **(F)**, contralateral volumes **(G)** or infarct ratios **(H)** among T2, DWI and TTC images which were analyzed by paired-sample *t*-test. [n = 11 in **(B–H)**].

To investigate the relationship between neuroimaging and pathology of experimental stroke, we compared MRI and TTC staining in infarcts of different extents. Figure 1A showed the images of TTC and MR at the same level of a rat brain after stroke, which had similar regions of infarcts. Quantitatively, both the direct infarct volumes and direct infarct ratios on T2 or DWI sequences of MRI showed strong correlations with the corresponding parameters on TTC staining (Figures 1B–E). Of note, the direct infarct volumes and infarct ratios on TTC staining were significantly larger than that on MRI, with larger differences in infarct volumes than in infarct ratios (the mean infarct volumes on TTC, T2, and DWI were 178.6, 130.7, and 123.7 mm^3^, respectively; *p*-value of TTC vs. T2 = 0.004; *p*-value of TTC vs. DWI = 0.007) (Figures 1F–H). The difference of infarct volumes between TTC and MRI was significantly correlated with the direct infarct volume on TTC (coefficient = 0.23, *p* = 0.008, 95% CI = 0.08–0.40) and edema-adjusted infarct volume on TTC (coefficient = 0.26, *p* = 0.040, 95% CI = 0.02–0.51), but not associated with ischemic duration (*p* = 0.717 by Kruskal-Wallis test). Therefore, infarct ratios instead of infarct volumes were used for presentation of infarct sizes in the subsequent analyses.

### Circling Behavior of Stroke Rats

The numbers of the rats with circling behavior after stroke according to occlusion time were shown in Table 2. Even circling gait was a common feature during occlusion period in tMCAO procedure, it became less prevalent (41%) after reperfusion. No rat had circling behavior after occlusion for 0.5 h, and the percentage of rats with circling behavior increased with prolonged occlusion time with a ceiling effect at occlusion for 1.5 h.

**Table 2:** The number of rats with circling behavior after stroke according to occlusion time.

**Circling, *n* (%)**	**Ischemic duration**					
	**0.5 h**	**0.75 h**	**1 h**	**1.5 h**	**2 h**	**3 h**
Discovery group	0 (0%)	1 (20%)	2 (29%)	4 (80%)	0 (0%)	2 (67%)
Validation group	0 (0%)	1 (20%)	7 (47%)	9 (64%)	2 (67%)	2 (67%)

*The percentages were calculated as (number of rats with circling in each occlusion time)/(number of rats in each occlusion time)*.

### The Relationship Between Ischemic Duration and Pathological Extent of Infarct

**Figure 2:**
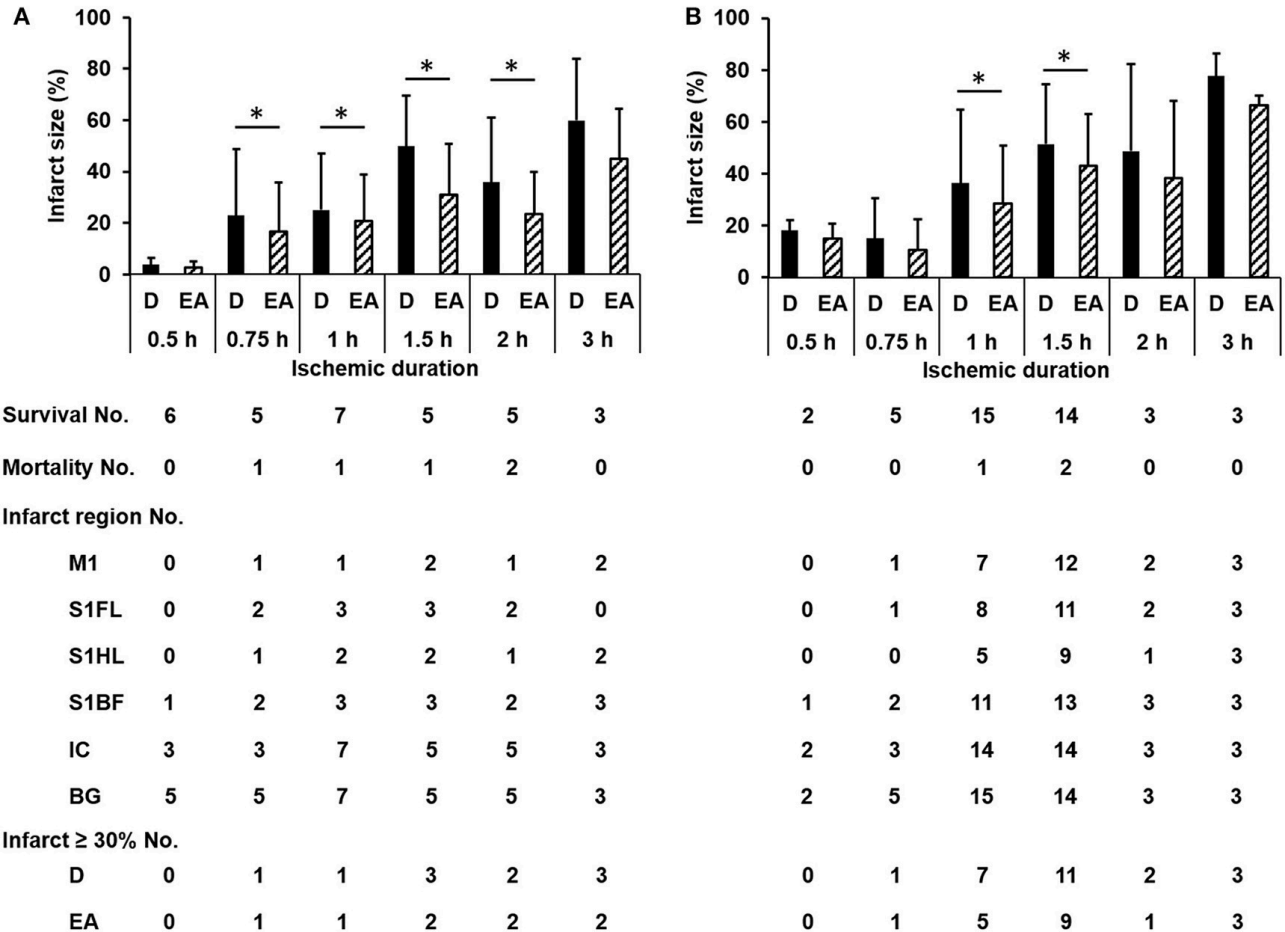
The numbers of survival, mortality, specific infarct regions and infarct sizes according to different ischemic durations in the discovery and validation groups. The infarct sizes differed significantly according to ischemic durations (discover group **(A)**: *p* = 0.001 and 0.004 for direct infarct size and edema-adjusted infarct size, respectively; validation group **(B)**: *p* = 0.008 and 0.006, respectively). Edema-adjusted infarct sizes (EA) were significantly different from the direct infarct sizes (D) within the same ischemic duration of 0.75 h to 2 h in the discovery group, and 1 h and 1.5 h in the validation group (*p* < 0.05). Basal ganglion (BG) and internal capsule (IC) were commonly involved in all ischemic durations. There was no difference about the distribution of the involved regions in M1, S1FL, S1HL, S1BF, IC, and BG according to ischemic durations in both groups (*p* = 0.996 and 0.994, respectively). M1, the primary motor cortex; S1FL, the forelimb region of the primary sensory cortex; S1HL, the hindlimb region of the primary sensory cortex; S1BF, the barrel field region of the primary sensory cortex; IC, internal capsule; BG, basal ganglion; D, direct infarct size; EA, edema-adjusted infarct size.

The numbers of survival and mortality, as well as the infarct sizes and involved regions according to different ischemic durations in the discovery and validation groups were shown in Figure 2. The mortality rate in each time group was 0/6, 1/6, 1/8, 1/6, 2/7, and 0/3 induced by ischemic duration 0.5, 0.75, 1, 1.5, 2, and 3 h in the discovery group, and 0/2, 0/5, 1/16, 2/16, 0/3, and 0/3, respectively in the validation group. The infarct sizes differed significantly according to ischemic durations (discover group: *p* = 0.001 and 0.004 by Kruskal-Wallis test for direct infarct size and edema-adjusted infarct size, respectively; validation group: *p* = 0.008 and 0.006, respectively). Combined the two groups together to compare the infarct sizes between contiguous ischemic durations, the infarct sizes were significantly different between ischemic duration 0.75 h and 1 h (*p* = 0.038 and 0.019 for direct infarct size and edema-adjusted infarct sizes, respectively, by Mann-Whitney *U* test), and between 1 and 1.5 h (*p* = 0.017 and 0.021 for direct infarct size and edema-adjusted infarct sizes, respectively). Ischemic duration between 2 and 3 h also induced a significant difference in direct infarct sizes (*p* = 0.039) but not edema-adjusted infarct sizes (*p* = 0.053). After pooling the two groups together, the two types of infarct sizes differed significantly in all ischemic durations (*p* < 0.05 by Wilcoxon matched-pairs signed-rank test), especially in 0.75–1.5 h (*p* < 0.01).

### Functional Scoring of Behaviors for the Pathological Extent of Ischemia: Prediction of Infarct Size

To ask whether each item of functional assessments could correspond to the pathological extent of brain ischemia, we analyzed the predictability of each subscore for edema-adjusted infarct size ≥30%, in the discovery group of 31 stroke rats (Table 3). With adjustment of ischemic duration, subscores 1 (spontaneous activity), 4 (climbing), 5 (body sensation), 6 (vibrissae sensation), and 7 (circling) were significantly correlated with edema-adjusted infarct size ≥30% (*p* = 0.036, 0.021, 0.021, 0.008, and 0.010, respectively).

**Table 3:** Prediction of infarct sizes and neuroanatomical locations by the Garcia subscores and a new subscore 7 (circling).

	**Unadjusted *p*-value**	**Adjusted *p*-value^a^**
**PREDICTION OF EDEMA-ADJUSTED INFARCT SIZE** **≥30%**		
Subscore 1	0.014^*^	0.036^*^
Subscore 3	0.038^*^	0.141
Subscore 4	0.008^*^	0.021^*^
Subscore 5	0.010^*^	0.021^*^
Subscore 6	0.005^*^	0.008^*^
Subscore 7	0.003^*^	0.010^*^
**PREDICTION OF INFARCT IN M1 REGION**		
Subscore 1	0.008^*^	0.020^*^
Subscore 4	0.009^*^	0.019^*^
Subscore 6	0.007^*^	0.010^*^
Subscore 7	0.003^*^	0.006^*^
**PREDICTION OF INFARCT IN S1FL REGION**		
Subscore 4	0.015^*^	0.011^*^
Subscore 6	0.035^*^	0.021^*^
**PREDICTION OF INFARCT IN S1HL REGION**		
Subscore 1	0.014^*^	0.031^*^
Subscore 4	0.008^*^	0.015^*^
Subscore 5	0.008^*^	0.017^*^
Subscore 6	0.004^*^	0.008^*^
Subscore 7	0.003^*^	0.007^*^
**PREDICTION OF INFARCT IN S1BF REGION**		
Subscore 3	0.002^*^	0.008^*^
Subscore 4	0.004^*^	0.011^*^
Subscore 5	0.008^*^	0.042^*^
Subscore 6	0.002^*^	0.006^*^
Subscore 7	0.029^*^	0.067

*The subscores unable for significant prediction were not shown*.

* *p-value < 0.05*.

a *adjusted with ischemic duration*.

*Definition of subscores: 1, spontaneous activity; 2, symmetry in the movement of four limbs; 3, forepaw outstretching; 4, climbing; 5, body sensation; 6, vibrissae sensation; 7, circling*.

*M1, the primary motor cortex; S1FL, the forelimb region of the primary sensory cortex; S1HL, the hindlimb region of the primary sensory cortex; S1BF, the barrel field region of the primary sensory cortex*.

**Figure 3:**
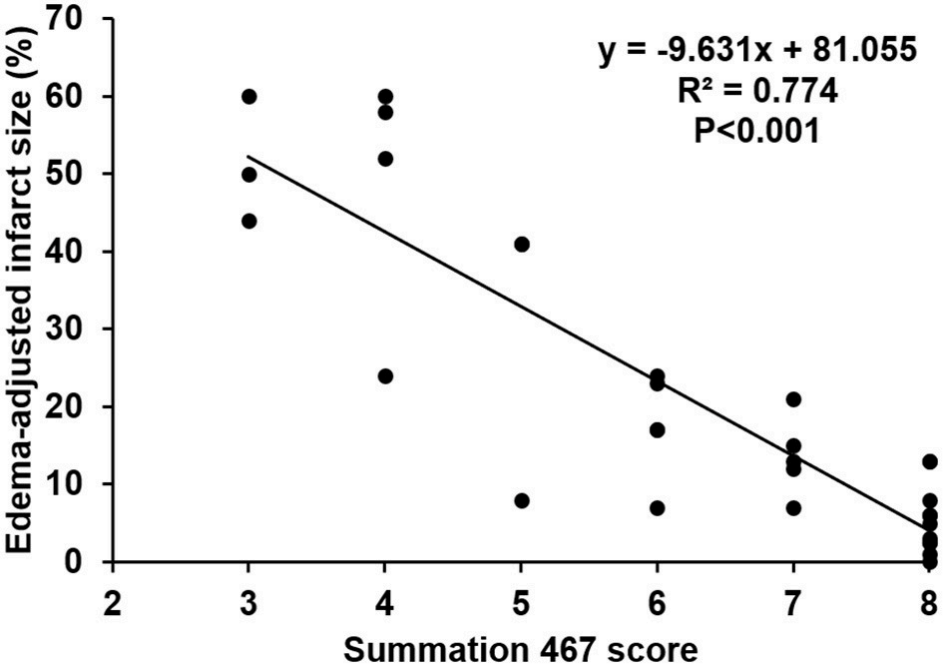
Linear correlation between the score of the summation (4,6,7) and edema-adjusted infarct size in the discovery group. Definition of subscores: 4, climbing; 6, vibrissae sensation; 7, circling (*n* = 31).

The scores of modified summations obtained by the combinations of these significant subscores were analyzed for the efficacy of predicting infarct size followed by comparison with the original Garcia score (Table 4). The original Garcia score, the scores of summation (4,5,6,7) (summation of subscore 4, 5, 6, and 7) and summation (4,6,7) could predict edema-adjusted infarct size ≥30% (*p* = 0.017, 0.013, and 0.016, respectively; AUC = 0.967, 0.970, and 0.976, respectively). The summation (4,5,6,7) score had similar prediction efficacy as compared with the original Garcia score (*p* = 0.887), and so did summation (4,6,7) score (*p* = 0.714). Furthermore, the summation (4,6,7) score had a linear correlation with edema-adjusted infarct size in the discovery group (*R*^2^ = 0.774, *p* < 0.001) (Figure 3).

**Table 4:** Comparison of the area under receiver operating characteristic curve (AUC) according to the scores of modified Garcia summations and the original Garcia score for predicting edema-adjusted infarct size ≥30%.

	***p*-Value of logistic regression model**	**AUC**	***p*-Value of AUC comparison ^a^**
**PREDICTION OF EDEMA-ADJUSTED INFARCT SIZE** **≥30%** ^b^			
Original Garcia score (1,2,3,4,5,6)	0.017^*^	0.967	
Summation (4,5,6,7) score	0.013^*^	0.970	0.887
Summation (4,6,7) score	0.016^*^	0.976	0.714

* *p-value < 0.05*.

a *Comparison of AUC of modified summation with the original Garcia score*.

b *Edema-adjusted infarct size (%) = (contralateral hemisphere volume—ipsilateral non-infarcted volume)/contralateral hemisphere volume*.

*N = 31*.

*Definition of subscores: 1, spontaneous activity; 2, symmetry in the movement of four limbs; 3, forepaw outstretching; 4, climbing; 5, body sensation; 6, vibrissae sensation; 7, circling*.

### Functional Scoring of Behaviors for Predicting Ischemia in Specific Brain Areas

To develop a new neuroanatomy-based scoring system which potentially reflects specific infarct brain areas, four neuroanatomical areas (primary motor cortex, M1; the forelimb region of the primary sensory cortex, S1FL; the hindlimb region of the primary sensory cortex, S1HL; and the barrel field region of the primary sensory cortex, S1BF) were chosen according to their functional relationship with these subscores. There was no difference about the distribution of the involved regions in M1, S1FL, S1HL, S1BF, internal capsule and basal ganglion according to ischemic durations in the discovery and validation groups (*p* = 0.996 and 0.994, respectively) (Figure 2). Basal ganglion and internal capsule were commonly involved in all ischemic durations.

The concomitantly involved cortical regions in infarcts were shown in Table 5. Infarct involving M1 was always accompanied with infarct in other regions (10/10 and 24/25 in the discovery and validation groups, respectively), while infarct in S1BF had lower probability of concomitantly involving other regions (13/17 and 27/33, respectively).

**Table 5:**
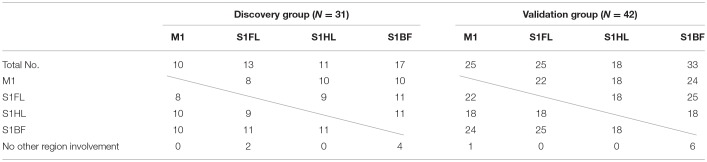
Concomitant involvement of other specific brain regions according to each specific brain region of infarct in the discovery and validation groups.

*M1, the primary motor cortex; S1FL, the forelimb region of the primary sensory cortex; S1HL, the hindlimb region of the primary sensory cortex; S1BF, the barrel field region of the primary sensory cortex*.

Next, we analyzed the predictability of each subscore for infarct in the 4 specific brain areas (M1, S1FL, S1HL, and S1BF) (Table 3). With adjustment of ischemic duration, subscores 1 (spontaneous activity), 4 (climbing), 6 (vibrissae sensation), and 7 (circling) showed significant prediction effect for infarct in M1 (*p* = 0.006–0.020). Subscores 4 and 6 could significantly predict infarct in S1FL (*p* = 0.011 and 0.021, respectively). Subscores 1, 4, 5 (body sensation), 6, and 7 had significant correlation with infarct in S1HL (*p* = 0.007–0.031). Significant predictors of infarct location in S1BF included subscores 3, 4, 5, and 6 (*p* = 0.006–0.042).

We then tested the hypothesis: whether combinations of these significant subscores could predict functionally correlated ischemic brain areas at the motor and sensory cortexes. The results were shown in Table 6. The original Garcia score failed to predict infarct in M1 (*p* = 0.995). The summation (1,4,6,7) score, designed by combination of the significant subscores, potentially could predict for infarct in M1 (*p* = 0.025, AUC = 0.970). The scores of summations (1,4,7), (1,6,7), and (4,6,7), simplified from the summation (1,4,6,7), also revealed significant correlation with infarct in M1 [(1,4,7): *p* = 0.003, AUC = 0.917; (1,6,7): *p* = 0.008, AUC = 0.964; (4,6,7): *p* = 0.028, AUC = 0.979]. For infarct in S1FL, the original Garcia score and the score of summation (4,6,7) showed significant prediction effect (*p* = 0.040 and 0.021; AUC = 0.750 and 0.786, respectively). The original Garcia score and the scores of summation (1,4,7), (1,6,7), and (4,6,7) all showed significant correlations with infarcts in S1HL or S1BF, and these modified scores were non-inferior to the original Garcia score in AUC comparison. The summation (4,6,7) score containing the components of motor, sensory, and gait pattern, had significant prediction of infarct locations in the primary cortex (M1) and sensory cortex corresponding to different body parts of the forelimb, hindlimb, and whiskers (S1FL, S1HL, and S1BF).

**Table 6:** Comparison of the area under receiver operating characteristic curve (AUC) according to the original Garcia score and the scores of modified summations for predicting infarct in the specific motor or sensory cortical regions.

	***p*-Value of logistic regression model**	**AUC**	***p*-Value of AUC comparison ^a^**
**INFARCT IN M1 REGION**			
Original Garcia score (1,2,3,4,5,6)	0.995	0.988	
Summation (1,4,6,7) score	0.025^*^	0.970	0.571
Summation (1,4,7) score	0.003^*^	0.917	0.221
Summation (1,6,7) score	0.008^*^	0.964	0.486
Summation (4,6,7) score	0.028^*^	0.979	0.727
**INFARCT IN S1FL REGION**			
Original Garcia score (1,2,3,4,5,6)	0.040^*^	0.750	
Summation (1,4,7) score	0.136	0.707	0.407
Summation (1,6,7) score	0.173	0.655	0.341
Summation (4,6,7) score	0.021^*^	0.786	0.186
**INFARCT IN S1HL REGION**			
Original Garcia score (1,2,3,4,5,6)	0.008^*^	0.938	
Summation (1,4,7) score	0.003^*^	0.886	0.400
Summation (1,6,7) score	0.004^*^	0.878	0.441
Summation (4,6,7) score	0.005^*^	0.927	0.593
**INFARCT IN S1BF REGION**			
Original Garcia score (1,2,3,4,5,6)	0.003^*^	0.872	
Summation (1,4,7) score	0.013^*^	0.826	0.470
Summation (1,6,7) score	0.010^*^	0.800	0.198
Summation (4,6,7) score	0.003^*^	0.899	0.390

* *p-value < 0.05*.

a *Comparison of AUC of modified Garcia summation with the original Garcia score*.

*M1, the primary motor cortex; S1FL, the forelimb region of the primary sensory cortex; S1HL, the hindlimb region of the primary sensory cortex; S1BF, the barrel field region of the primary sensory cortex*.

*Definition of subscores: 1, spontaneous activity; 2, symmetry in the movement of four limbs; 3, forepaw outstretching; 4, climbing; 5, body sensation; 6, vibrissae sensation; 7, circling*.

### Validation of the New Scoring System to Predict Ischemic Brain Areas and Infarct Size

We further analyzed the predictability of the summation (4,6,7) score for infarcts in specific brain areas and pathological extent in another group of 42 rats with stroke (the validation group). The cut-off values of the original Garcia score and the summation (4,6,7) score for predicting (1) infarct in specific brain areas of the motor cortex (M1) or sensory cortex (S1FL, S1HL, S1BF) and (2) edema-adjusted infarct sizes ≥30% were determined, respectively by Youden's index in the discovery group, then these values were tested for sensitivity, specificity, positive predictive value (PPV) and negative predictive value (NPV) in the validation group (Table 7). The original Garcia score had no significant correlation with infarct in M1 in the discovery group, thus its cut-off value was not shown. The predictive efficacy for edema-adjusted infarct size ≥30%, or infarct in S1HL or S1BF was similar between the Garcia score and the summation (4,6,7) score (*p* = 0.317–1.000 by McNemar test).

**Table 7:** Validation of prediction for infarct size and specified infarct locations by the original Garcia score and the summation (4,6,7) score.

	**Cut-off**	**Sen**	**Spec**	**PPV**	**NPV**	***p*-Value^a^**
**PREDICTION OF EDEMA-ADJUSTED INFARCT SIZE** **≥30%**						
Original Garcia score (1,2,3,4,5,6)	12	0.96	0.78	0.85	0.93	1.000
Summation (4,6,7) score	6	1.00	0.70	0.73	1.00	
**PREDICTION OF INFARCT IN M1 REGION**						
Original Garcia score (1,2,3,4,5,6)						(Inability)^*^
Summation (4,6,7) score	6	0.88	0.76	0.85	0.81	
**PREDICTION OF INFARCT IN S1FL REGION**						
Original Garcia score (1,2,3,4,5,6)	15	1.00	0.29	0.68	1.00	0.500
Summation (4,6,7) score	8	1.00	0.12	0.41	1.00	
**PREDICTION OF INFARCT IN S1HL REGION**						
Original Garcia score (1,2,3,4,5,6)	12	1.00	0.63	0.67	1.00	0.317
Summation (4,6,7) score	6	1.00	0.67	0.69	1.00	
**PREDICTION OF INFARCT IN S1BF REGION**						
Original Garcia score (1,2,3,4,5,6)	15	0.97	0.44	0.86	0.80	1.000
Summation (4,6,7) score	7	0.85	0.89	0.97	0.62	

* *The summation (4,6,7) score was superior to the original Garcia score because the latter one could not predict the specified character and was thus labeled as (inability)*.

a *Comparison between the summation (4,6,7) score with the original Garcia score by McNemar test*.

*Sen, sensitivity; Spec, specificity; PPV, positive predictive value; NPV, negative predictive value; M1, the primary motor cortex; S1FL, the forelimb region of the primary sensory cortex; S1HL, the hindlimb region of the primary sensory cortex; S1BF, the barrel field region of the primary sensory cortex*.

Since the summation (4,6,7) score, composed of neuroanatomy-based functional subscores, potentially predicted specific brain areas of ischemia and the pathological extent of infarct, it was designated as the modified Garcia score including motor functions (climbing and circling) and sensory function (vibrissae sensation).

### Neuroanatomy-Based Validation of Summation (4,6,7) Score According to Infarcts in These Functionally Related Cortical Areas

**Figure 4:**
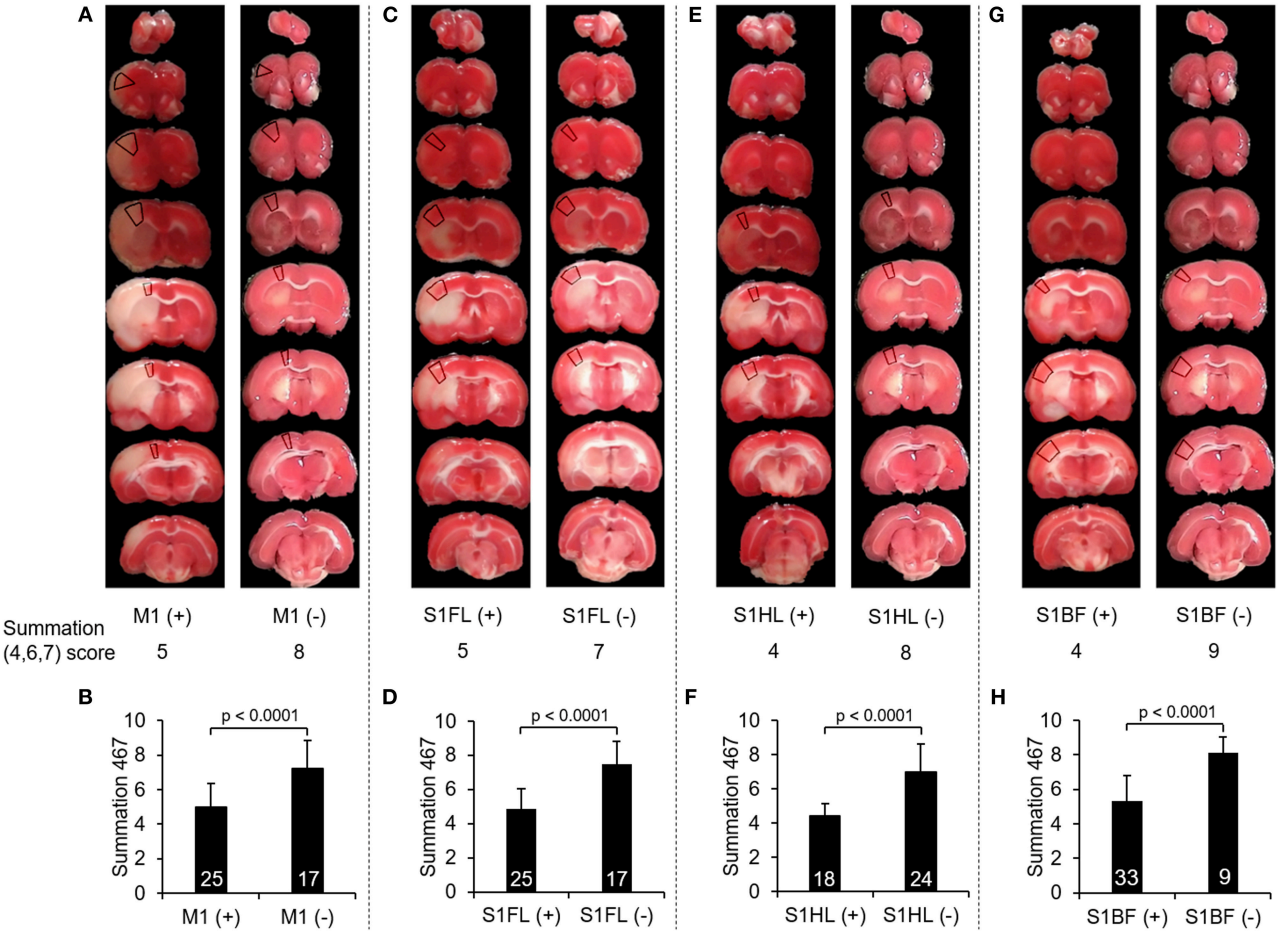
Comparison of the scores of summation (4,6,7) according to infarcts in one of these neuroanatomical areas, including M1, S1FL, S1HL, and S1BF. **(A)**, **(C)**, **(E)**, and **(G)** showed examples of TTC images with infarcts and without infarcts in M1, S1FL, S1HL, and S1BF, respectively, and the marked regions on TTC images indicated the target neuroanatomical areas. Their scores of summation (4,6,7) were listed below each TTC images. **(B)**, **(D)**, **(F)**, and **(H)** showed the comparisons of the summation (4,6,7) scores with infarcts to those without infarcts in M1, S1FL, S1HL, and S1BF, respectively, in 42 stroke rats. The rats with infarcts in one of these areas had significantly lower score in the summation (4,6,7) compared to those without infarcts in the corresponding area. Definitions of subscores: 4, climbing; 6, vibrissae sensation; 7, circling. Abbreviations: M1, the primary motor cortex; S1FL, the forelimb region of the primary sensory cortex; S1HL, the hindlimb region of the primary sensory cortex; S1BF, the barrel field region of the primary sensory cortex.

To further validate whether there would be any difference in the summation (4,6,7) score according to the involvement of these functionally correlated cortical areas, we compared the scores of summation (4,6,7) in the validation group of stroke rats with M1 infarcts to those without M1 involvement, and so did other specific regions including S1FL, S1HL, and S1BF. In all of these selected neuroanatomical areas, the rats with involvement at one of these regions had significantly lower scores in the summation (4,6,7) than those without such involvement (all *p* < 0.0001) (Figure 4).

### Comparison of Efficiency Between the Modified Garcia Score and the Original Garcia Score

In the 10 rats evaluating for the original and modified Garcia scores upon serial time points at 24, 72, and 120 h, there was no difference between any two time points in either score (*p* = 0.211–1.000).

To understand whether there would be additional advantages of the modified Garcia score, we analyzed two factors: (1) examination time and (2) inter-rater reliability in 13 rats. The modified Garcia score required less examination time than the original Garcia score (132.1 ± 60.7 vs. 447.6 ± 61.1 s, *p* < 0.001). Moreover, the modified Garcia score had a higher degree of agreement than the original Garcia score between two independent observers. Inter-rater reliability of the original Garcia score had only 69.2% agreement, with kappa 0.65, indicating “substantial agreement” (McHugh, 2012). In contrast, inter-rater reliability of the modified Garcia score increased up to 84.6%, with kappa 0.80, which indicated “almost perfect agreement” (McHugh, 2012).

## Discussion

The study proposed a potential functional scoring system of rat behaviors after tMCAO according to structural involvement of infarcts by (1) deleting non-significant subscores, (2) adding a new subscore of circling, and (3) combining the subscores according to the predictability of each subscore for the specific brain areas and the pathological extent of infarcts, in order to increase the prediction strength of the modified Garcia score. Specifically, in the discovery group of stroke rats, summation (4,6,7) score had the potential to predict infarct sizes and ischemic neuroanatomical areas, respectively. We further applied these analyses to the validation group of stroke rats to compare the prediction ability of this modified scoring version vs. the original Garcia score. Based on these analyses, we designed this neuroanatomy-based functional scoring of rat behaviors including (1) motor functions (climbing and circling) and (2) sensory function (vibrissae) to predict ischemic brain areas and infarct sizes. Furthermore, the modified Garcia score consumed less examination time and had better inter-rater reliability than the original Garcia score. Importantly, these behavioral subscores probably reflect underlying neuroanatomical functions. For example, the motor functions of climbing and circling require the motor cortex M1 and the sensory cortex of forelimb and hindlimb regions. The sensory subscore of vibrissae requires the integrity of the barrel field region of the sensory cortex and motor function to turn head.

Although a few previous behavioral tests correlated with infarct size, some of them were only able to predict infarct size within a certain range of size or predict infarct areas in a defined brain region (Bederson et al., 1986; Ishibashi et al., 2003, 2004). There was a lack of systemic evaluation of a behavioral test to predict infarct size with a cut-off point. Predicting of infarct size ≥30% is potentially important because it indicates that the infarct size is larger than average size (Conn, 2017). The score of summation (4,6,7) (climbing, vibrissae sensation, and circling, respectively) predicted edema-adjusted infarct size ≥30% in the tMCAO rats after different durations of ischemia that encompassed a wide range of infarct size, indicating its application potential to a wide range of infarct size in this stroke model. In summary, this score of summation (4,6,7) was probably able to predict infarct size.

Infarct of a specific neuroanatomical area is theoretically manifested with specific functional deficits documented by corresponding behavioral tests. Previous reports demonstrated that certain behavioral tests correlated with regional infarct volumes of specific brain areas (Ishibashi et al., 2003, 2004). However, there was a lack of systematic studies using a single behavioral test to predict the involvement of infarcts in several functionally correlated brain regions. Furthermore, most of these reported tests required considerable time that might make stroke rats fatigued or habituated, and some even required specific medication for test (Ishibashi et al., 2003, 2004). Less items of subscores in a behavior test may also make training of raters more easily. The inter-rater differences in the Garcia subscores other than subscore 4, 6, and 7 may contribute to lower inter-rater agreement in the original Garcia score, which might require more intensive training of raters. The present study designed a new behavioral score of summation (4,6,7) (climbing, vibrissae sensation, and circling, respectively), which probably could predict infarct in functionally relevant sensorimotor cortical regions, including M1, S1FL, S1HL, and S1BF. The original Garcia score was unable to predict infarct in M1, but the score of summation (4,6,7) could predict the region successfully, as well as S1FL, S1HL, and S1BF. Therefore, the summation (4,6,7) score is superior to the original Garcia score in the prediction of infarct at functionally-correlated regions in the motor and sensory cortexes.

Circling behavior is a common manifestation of rats after tMCAO, especially in those during acute stage of severe stroke (Reglodi et al., 2003). It has been enrolled into several single or composite behavioral tests (Bederson et al., 1986; Longa et al., 1989; Shohami et al., 1995), but not in the original Garcia score. Moreover, Bachour et al. reported that Garcia score and Longa score had low correlation in determining neurological changes after stroke (Bachour et al., 2016). The two scores might evaluate different neurological components and combination of them might improve the detection ability. Besides, we observed that even though all of the rats had circling behavior during occlusion period of tMCAO, only 41% of rats persisted to have circling gait after reperfusion. More rats had circling behavior with increased occlusion time, but had a ceiling effect at 1.5 h, indicating the presence of circling behavior might correlate with stroke severity. Since circling is a prominent behavior and easy to be evaluated, we added this item into modified Garcia scoring system, which showed benefits in predicting (1) ischemic brain areas in the motor cortex of M1 and sensory cortex of S1HL and (2) pathological extent of infarct ≥30%. Circling behavior is caused by limb weakness, indicating that the lesion involves the motor pathway, from the motor cortex to the internal capsule in the middle cerebral artery territory. In all of the rats with circling behavior in the discovery and validation groups (*n* = 30), 22 (73%) rats had infarcts in the M1 region and all (100%) had infarcts in the internal capsule. Thirty rats (47%) had circling behavior in those with infarcts in internal capsule, while none had circling gait (0/8) in those with infarcts not involving this region. Therefore, internal capsule is another possible lesion site in addition to M1 for presentation of circling behavior.

The subscore 2, 3, and 7 all evaluate motor asymmetry, but they may represent different severities of hemiparesis. The subscore 2 and 3 could be abnormal even in mild and moderate weakness, while circling behavior usually indicated more severe weakness. In all of the rats in the discovery and validation groups, 50% of the rats with abnormal subscore 2 had circling behavior, and 62% of the rats with abnormal subscore 3 had circling gait. Since the predictive targets of this study indicated relatively large infarct extent, it was reasonable that subscore 2 and 3 could not arrive statistical significance but circling behavior could.

In this study, the infarct sizes were larger in ischemia for 1 h compared with those for 0.75 h, and larger in ischemia for 1.5 h compared with 1 h, which was compatible with the findings in some previous reports (Popp et al., 2009; Lee et al., 2014). However, we noted that the infarct sizes had a ceiling effect in the ischemic duration longer than 1.5 h, and also had no difference between ischemia for 0.5 and 0.75 h. Some other studies also showed that infarct volumes were not necessarily larger in longer ischemia. Ingberg et al. reported that infarct sizes were similar between tMCAO for 45 and 60 min when evaluated at 24 h in Wistar rats (Ingberg et al., 2018). Du et al. reported that the infarct sizes after 30 and 90-min tMCAO in Long-Evans rats were similar when evaluated at 2 weeks (Du et al., 1996), and Hattori et al. also found that infarct sizes evaluated after 2 weeks had no difference between 60 and 90-min tMCAO in mice (Hattori et al., 2000). This discrepancy may be due to variable infarct sizes in the same occlusion time, different animal species and strains, and different timing of evaluation (Balkaya et al., 2013).

There was a significant difference of infarct volumes between MRI and TTC in this study, and the difference significantly correlated with direct infarct volume and edema-adjusted infarct volume on TTC, which indicated that the rats with larger infarct volumes would have larger volume differences of infarcts between MRI and TTC. Post-stroke edema may also contribute to this difference because the direct infarct volume seemed more significantly correlated with the volume difference than edema-adjusted infarct volume. It was possible that the edematous portion of brain in stroke regions was more susceptible to aggravated edema after immersed in TTC solution.

The different slice thickness on MRI (1 mm) and TTC (2 mm) may also contribute to the difference in accuracy in estimating the volume by these two methods, making larger estimation error in TTC method. Besides, thinner slices have higher accuracy in the estimation for volumes in MRI studies, and the slice thickness was suggested to be less than a fifth of anticipated diameter of lesions (Firbank et al., 1999). Another study showed that slice thickness of 1, 3, and 5 mm did not have significant difference in calculating the volume of human hippocampus (Laakso et al., 1997). Bradley et al. found that detection ability for high-contrast lesions on MRI was determined by the presence of a gap between slices instead of slice thickness (Bradley and Glenn, 1987). The infarct lesions in rat brains were usually high-contrast and not small, thus slice thickness may not necessarily affect the measurement accuracy on MRI. In addition, slice thickness of 1 mm on MRI is commonly used for rat stroke studies (Choi et al., 2018; Yu et al., 2018).

The concomitant infarct regions of these specific brain areas was interesting. In the tMCAO model, M1 involvement was always accompanied with other regions including S1BF, S1HL, or S1BF (100–96%). However, S1BF involvement in infarct had lower probability accompanying with other regions (82–76%). In their neuroanatomical locations, M1, S1FL, and S1HL regions are in direct contact with each other, while S1BF region is only near S1FL but has a gap between them. The anatomical distribution of the 4 regions may contribute to these findings. In addition, these findings also indicated that M1 infarct in this stroke model probably accompanied with infarct of other somatosensory regions, which may present with impairment in certain motor and sensory functions.

There are several limitations in this study. The first limitation is the small number of rats in each ischemic duration. This study mainly focused on the correlation of behavior scores with infarct size or infarct location. Nevertheless, the consistent findings in the discovery group and the validation group indicated that the small number of rats in each ischemic duration might not strongly affect the results and suggested the robustness of correlation analysis. The second potential limitation is the timing of behavioral evaluation, which might influence the behavioral performance due to compensation. However, we found that the original Garcia score and summation (4,6,7) score were stable during the first 5 days after stroke. Other study found that even though the gait of stroke rats had compensations in stance duration and stride length, these rats had relatively mild gait disturbance compared with their severe sensorimotor deficits (Parkkinen et al., 2013). Taken together, compensation might not significantly affect the performance in the original and modified Garcia scores within 5 days. The third potential limitation is the small sample of rats used to correlate MRI with TTC staining. This was because TTC staining has been a widely used method to evaluate infarct size in rats (Benedek et al., 2006) and MRI study is not always available. Thus, we used TTC staining to evaluate the pathological extent of brain in all of the rats in the discovery and validation groups, and checked a small number of the rats by MRI to evaluate its correlation with TTC staining. The fourth potential limitation is the complexity of circuits between different cortical regions and cortical-striatal regions, as well as potential functional overlapping of cortical areas and possible reorganization of the circuits after ischemic injury, making it possibly difficult to predict infarct in a specific brain region by a behavior test. Two additional factors further affected the relationship between behavior tests and infarct regions. One factor was the complex relationship between the behavior scores and functions: subscore 4 (climbing) tested motor functions and joint position sensations of four limbs, and subscore 6 (vibrissae sensation) evaluated vibrissae touch sensation and motor function to turn head. Another factor was that infarct in one of the four sensori-motor cortical regions usually had concomitant infarct in other regions in this model, and all of the affected regions might potentially affect the behavioral performance. These complex factors may contribute to the imperfect prediction for infarct in M1 region by the original Garcia scores, and the modified Garcia score had better prediction efficacy through deleting non-significant subscores and adding subscore 7 (circling). Although the behavioral tests in this study could not fully reveal the condition of these related circuits, they potentially had an association with the involvement of certain important regions within these circuits.

## Conclusions

In conclusion, in order to predict the ischemic brain areas and the pathological extent of infarcts, this study designed a new behavioral scoring system by systematic analysis of the original subscores of the Garcia system and adding a new subscore of circling. We proposed this neuroanatomy-based functional examination of behavioral score consisting of (1) motor functions of climbing and circling, and (2) sensory functions of vibrissae, which was probably able to predict ischemic changes in the motor cortex of M1 and sensory cortex of relevant body parts for applications of experimental stroke studies. This modified score had better prediction ability than the original Garcia score for infarct involving M1. We further demonstrated that the modified Garcia score had improved efficiency: (1) less examination time and (2) better inter-rater reliability than the original Garcia score.

## Data Availability Statement

The raw data supporting this study is available in the Supplementary Data Sheet.

## Author Contributions

S-JY and S-TH contributed conception and design of the study. S-JY organized the database. S-JY and C-LC performed the statistical analysis. S-JY wrote the first draft of the manuscript. All authors contributed to manuscript revision, read and approved the submitted version.

### Conflict of Interest Statement

The authors declare that the research was conducted in the absence of any commercial or financial relationships that could be construed as a potential conflict of interest.
