# Supplementary material for: Neuroanatomy- and Pathology-Based Functional Examinations of Experimental Stroke in Rats: Development and Validation of a New Behavioral Scoring System
Source: Front Behav Neurosci. 2018 Dec 18;12:316. doi: 10.3389/fnbeh.2018.00316 (PMC6305474; doi:10.3389/fnbeh.2018.00316)
Supplement: Supplementary file 1 [file Table_1.docx]

**Supplementary Material**

**Neuroanatomy- and Pathology-Based Functional Examinations of Experimental Stroke in Rats: Development and Validation of a New Behavioral Scoring System**

Shin-Joe Yeh, Sung-Chun Tang, Li-Kai Tsai, Jiann-Shing Jeng, Chi-Ling Chen and

Sung-Tsang Hsieh*

***Correspondence:** Dr. Sung-Tsang Hsieh, E-mail: shsieh@ntu.edu.tw

**1 Supplementary Tables**

**Supplementary Table 1. Review of previous behavioral tests for predicting size and neuroanatomical locations of infarcts in rodent stroke models**

| **Behavioral tests** | **Score Type** | **Motor** | **Sensory** | **Predicting infarct size** | **Predicting infarct location** |
| --- | --- | --- | --- | --- | --- |
| Bederson score (Bederson et al., 1986) | Simple score | (+) | (-) | Infarct size at 24 hours | (-) |
| An eight-point rating scale (Rogers et al., 1997) | Simple score | (+) | (-) | Infarct volumes (total, cortical, or striatal) at 24 hours | (-) |
| Neuro-score (Zausinger et al., 2000) | Simple score | (+) | (-) | Infarct volumes (total and cortical) on day 3 and 7 | (-) |
| Sensorimotor integration test (Grabowski et al., 1993) | Composite score | (+) | (+) | Correlation of the score at 1 and 2 months with total infarct size at 3 months | (-) |
| Neurological score (Yonemori et al., 1996) | Composite score | (+) | (+) | Correlation of the score at 2 months with total infarct size at 3 months | (-) |
| Clark focal score (Wen et al., 2017) | Composite score | (+) | (+) | Infarct size at 24 hours | (-) |
| Staircase test (Grabowski et al., 1993) | Task-specific score | (+) | (-) | Correlation of the score at 1, 2 and 3 months with infarct size at 3 months | (-) |
| Elevated body swing test (Ishibashi et al., 2003) | Simple score | (+) | (-) | Regional infarct volume in the primary motor cortex | Regional infarct volume in the primary motor cortex |
| Adhesive removal test (Ishibashi et al., 2003) | Task-specific score | (+) | (+) | Regional infarct volume in the sensory cortex (forelimb area) | Regional infarct volume in the sensory cortex (forelimb area) |
| T-maze test (Ishibashi et al., 2003) | Task-specific score | (+) | (-) | Total infarct volume and regional infarct volume in the primary visual cortex | Total infarct volume and regional infarct volume in the primary visual cortex |
| Methamphetamine-induced circling behavior (Ishibashi et al., 2004) | Simple score | (+) | (-) | Regional infarct volume in the striatum | Regional infarct volume in the striatum |

**References**

Bederson J.B., Pitts L.H., Tsuji M., Nishimura M.C., Davis R.L., Bartkowski H. (1986). Rat middle cerebral artery occlusion: evaluation of the model and development of a neurological examination. Stroke. 17, 472-476.

Grabowski M., Brundin P., Johansson B.B. (1993). Paw-reaching, sensorimotor, and rotational behavior after brain infarction in rats. Stroke. 24, 889-895.

Ishibashi S., Kuroiwa T., Endo S., Okeda R., Mizusawa H. (2003). Neurological dysfunctions versus regional infarction volume after focal ischemia in Mongolian gerbils. Stroke. 34, 1501-1506.

Ishibashi S., Kuroiwa T., Katsumata N., Yuan S.L., Endo S., Mizusawa H. (2004). Extrapyramidal motor symptoms versus striatal infarction volume after focal ischemia in Mongolian gerbils. Neuroscience. 127, 269-275.

Rogers D.C., Campbell C.A., Stretton J.L., Mackay K.B. (1997). Correlation between motor impairment and infarct volume after permanent and transient middle cerebral artery occlusion in the rat. Stroke. 28, 2060-2065.

Wen Z., Xu X., Xu L., Yang L., Xu X., Zhu J., et al. (2017). Optimization of behavioural tests for the prediction of outcomes in mouse models of focal middle cerebral artery occlusion. Brain Res. 1665, 88-94.

Yonemori F., Yamada H., Yamaguchi T., Uemura A., Tamura A. (1996). Spatial memory disturbance after focal cerebral ischemia in rats. J Cereb Blood Flow Metab. 16, 973-980.

Zausinger S., Hungerhuber E., Baethmann A., Reulen H., Schmid-Elsaesser R. (2000). Neurological impairment in rats after transient middle cerebral artery occlusion: a comparative study under various treatment paradigms. Brain Res. 863, 94-105.
